# 

**Published:** 1892-03-12

**Authors:** 


### To Our Readers.

On and from the issue of April 2nd, 1892, The Hospital will be permanently enlarged, important features will be introduced, and the price raised from one penny to twopence. Those of our readers who like to subscribe direct, by sending their subscriptions to the Publisher, at the Office, 140, Strand, W.C., will be supplied with The Hospital every week, post free, for 8s. a year; 5s. for half a year; or 2s. 6d. for three months. All subscriptions will be payable in advance.

**Notices of Births, Deaths, and Marriages,** are inserted in this column at a charge of 2s. 6d. for an announcement not exceeding four lines.

### NOTICE TO CORRESPONDENTS.

All MS., Letters, Books for Review, and other matters intended for the Editor should be addressed The Editor, The Lodge, Porchester Square, London, W.

The Editor cannot undertake to return rejected M.S., even when accompanied by stamped directed envelope.

### GENERAL CHARITIES.

[This Column is devoted to an account of work of charitable institutions other than Medical Charities. The Editor will be glad to receive reports or news of work at 140, Strand. *Philanthropy* should be plainly written on the envelope.]

THE Bishop of Worcester has become a vice-president of the Waifs and Strays' Society.

MR. GEORGE NEWNES, M.P., will preside at the annual dinner of the Asylum for Fatherless Children on April 6, and that of the Royal Hospital for Incurab'e , to be he'd on May 18.

AT the recent council meeting of the Children's Happy Evenings Associations, Susan, Countess of Malmesbury, the Countess of Iddesleigh, and Lady George Hamilton were elected to serve on the Executive Committee.

The Waifs and Strays Society has received a bequest of £1,100 for the Permanent Home for Crippled Children, and one of £250 for the Walsham Farm Home, which it is hoped will be established soon. Amongst the children for whom homes have been provided are those of the man Miller, who was sentenced recently to twenty years' penal servitude for the murder of his wife.

A NEW building for the Industrial Institution, the Working Boys' Home and Harding Technical School, has been opened in Lord Edward Street, Dublin. The home was established fifteen years ago, as a home for boys of good character earning small wages in Dublin. Up to the present time some five hundred and fifty boys have received the benefits of the institution, and many of them have remained in the home as long as seven years. The home which requires an annual sum of £800 for its maintenance is supported to a great extent by contributions from the boys, but voluntary contributions are also required to meet current expenses.

The Duke of Edinburgh made a highly satisfactory statement at the recent meeting of the **ROYAL SAILORS' HOME** in Devonport, to the effect that he has been in Devonport eighteen months, and has during that time never seen anything to complain of, so far as insobriety is concerned, in the conduct of the seamen of the fleet in the towns of Plymouth, Devonport, and Stonehouse. In his opinion such institutions as sailors' homes have done an immense deal to bring about such a satisfactory state of affairs. There seems no reason to doubt the correctness of this opinion, and it is to be hoped that these institutions will never be allowed to drop for want of funds.

Lovers of animals will be glad to hear that the Naples Anti-Cruelty Society has for some time past been doing good work. Naples has always had an unenviable notoriety as a hotbed of cruelty to animals, and how much needed the work of the society is is evinced by the fact that nearly forty thousand cases of ill-treatment were prevented or punished during the past year. As little or no assistance is forthcoming from Neapolitans, who are too apathetic to take interest in such work even if they have the inclination, the Society has to look to England for support, in need of which it now stands. Those who are willing to support this good work are invited to send donations to Miss P. H. Johnston, The Beeches, Carlisle, who acts as Hon. Treasurer in England.

Miss Frances Ashdown makes a further appeal for help on behalf of the Church Extension Association, 27, Kilburn Park Road, N.W., during the cold weather of the winter. There are many good works carried on by the Association, but they cannot be continued unless funds are forth coming, and now is not the time to discontinue work amongst the poor and starving. The Association takes under its care numerous children in Kilburn and other parishes who have never had enough wholesome food to eat or comfortable clothes to wear. For six shillings one hundred of these poor mites can be fed with soup, bread and milk, and pudding, but parents who can afford it have to give a half-penny towards the child's meal.

Of recent years labour and poverty have attracted a good deal of public attention, though not more than the importance of the problems presented for solution by both deserves. It is only since public interest has been so keenly aroused that philanthropic effort can be said to have been organised on a practical basis. Evidence of what good can be done by systematic organisation and thoroughness can be gleaned by a perusal of two publications, entitled respectively, "Seventh Annual Report of the Egham Free Registry for the Unemployed" and "Second Annual Report of the North Kensington Friendly Workers among the Poor." The work of the Egham Registry might with advantage be initiated in other parishes, and much credit reflects on Mr. N. L. Cohen for the practical manner in which he took to heart the object lesson presented to him by a man whom he came across in search of work after a wearisome tramp, which he might have got in his own parish had there been a labour registry there. The North Kensington Friendly Workers aim at getting to know the condition of all the people in a mapped-out district who are likely at any time to need assistance. The means employed are first to tabulate the people and their condition, then to enlist the services of those who will take an oversight of a few houses and be ready to help in emergencies. According to the report, "It has from the commencement been the fundamental aim of the society to do its work, not by seeking out faults in the poor and thereby excusing itself from holding out a helping hand to them, but, on the contrary, by allowing for and appreciating the good that is in them."

### SCRAPS AND GLEANINGS.

THE Working Men's Hospital collections in Leeds have increased from £200 to £5,000.

PAYMENTS by patients to the Provident Branch of Southampton Dispensary for 1891 amount to £500, half the total annual income.

THE scheme started by Dr. G. E. Macdonald, at Islip, in America, to establish a farm for the employment of insane patients, has answered admirably.

YET another cure for influenza hails from America, in the form of asafœtida which is said to be an unfailing specific if administered in four grain pills four times a-day.

IN a Bohemian village recently a couple were married on the same day that the bridegroom's parents celebrated their silver wedding and his grandparents their golden wedding.

TWENTY-SIX thousand, five hundred pounds has been subscribed to the Russian Famine Fund in addition to £1,000 received from Lord Monkswell and the Hon. G. Coleridge's committee.

THE fifth annual report of the Aberdeen Royal Infirmary shows that 2,052 patients were treated in the past year. The average number of beds occupied daily was 155, and the cost per bed was £39 18s. 5d.

ONE thousand and ninety-eight pounds has been raised as a memorial to the late Rev. H. White, chaplain to the Savoy Chapel. A portion will be devoted to the endowment of a bed in King's College Hospital.

THE Bath Eye Infirmary has reached it eightieth anniversary. The annual report states that 279 in-patients and 1,733 out-patients were treated in the past year. The institution is in a prosperous condition.

THE late Mr. J. H. Agnew, of Dywood Hall, Manchester, has bequeathed £1,000 to the Pendleton General Hospital and Dispensary, and £1,000 to Owen's College, Manchester, to found a Medical Scholarship.

THE annual meeting of the Westminster Ophthalmic Hospital, Sir R. Alcock, presiding, was held on the 3rd inst. 9,608 patients were treated, being an increase of 436. The income was £2,655 and the expenditure £2,235.

IN the recent half-yearly report of the Medical Officer of Health for the Port of London the value of continuous sanitary inspection is shown—out of 7,783 vessels inspected only 2.5 per cent. required cleansing.

THE number of patients who attended the Brighton, Hove, and Preston Dispensary during 1891 was 13,988, including 6,650 visited at their own homes. The total receipts amounted to £2,788 8s. 8d., and the expenditure to £2,623 12s. 7d.

THE annual report of the Seamen's Hospital states that 13,510 patients (the largest number since the foundation of the Charity) were treated at the society's various institutions during the year. The expenditure has exceeded the income by £2,479.

IT is proposed to endow a bed at the cost of £500 in the Dublin National Children's Hospital in memory of the late Duke of Clarence. The hospital was opened by His Royal Highness in 1887. The existing debt of £3,300 is now entirely cleared off.

A SINGULAR case of death is reported from the gnawing of a rat at Bow. An infant was in its cradle when a large rat, supposed to have escaped from the street, seized hold of the little one's nose. The poor child never rallied from the shock and died on Saturday.

SIR WILFRED LAWSON presided at the annual meeting of the Cumberland Infirmary. The income for the past year amounted to £4,287, being an increase of £823 on that for 1890, and the expenditure was £3,788. Payments by patients again exceeded £160.

THE Managing Committee of the Grimsby and District Hospital are to be congratulated on the result of their efforts to pay off the existing debt of £1,400 at the annual meeting. The Hon. Secretary announced the hospital to be free of all liabilities, with a substantial balance in hand for the coming year.

AMONGST the recent subscriptions received towards the New Infirmary at Lancaster, are £500 from Mr. Albert Greg, £500 anonymous, and £100 from Mrs. Peacock. The total receipts amount to £6,644, and the Committee appeal for further funds to enable them to commence building operations without delay.

WE are glad to be able to report on the success of the Stanmore Cottage Hospital, which was opened on September 24th, 1890. From that date up to December 31st, 1891, 104 in-patients were admitted. The Hospital contains seven beds and one cot. Payments by patients vary from 3s. 6d. to 10s. 6d. per week.

IN presiding at the annual meeting of the Children's Hospital, Belfast, the Marquis of Londonderry announced that the institution is in a very satisfactory condition. The year commenced with a treasurers' balance of £483 9s. 10d. The convalescent home has been found an invaluable adjunct to the hospital.

THE Governors of the Nottingham and Notts Convalescent Homes have decided to purchase houses at Skegness and Donithorne for the establishment of convalescent homes. The cost of purchase (to include alterations and furnishing) has been defrayed by the munificence of Colonel Seeley, who contributes £12,000 for the purpose.

AT the annual meeting of the Dumfriesshire and Galloway and Royal Infirmary it was stated that the total number of in-patients, 507, was the largest number ever treated in the Infirmary. The out-patients numbered 3,050. The average time during which patients were under treatment was 25·2 days, compared with 28·68 days in the previous year.

THE Scarisbrick Trustees have granted a free gift of a site of five acres of land for the New Southport Infirmary. The report of the present infirmary for the past year shows that 331 in-patients and 2,122 out-patients were treated. The total income amounted to £2,195 13s. 11d., and the disbursements to £2,213 8s., showing a debit balance of £17 14s. 1d.

THE South Coast Medical and Surgical Home, Clarendon Road, Southsea, partly supported by patients paying £1 1s. and 12s. 6d. per week appeals for funds to carry on its work. Heavy expenses have been incurred in moving to new and more commodious quarters. Seventy-nine patients were received during the past year, and the balance of the reserve fund stands at £50.

### THE STUDENT OF MEDICINE.

[All communications for this Supplement should be addressed to THE EDITOR of THE HOSPITAL, at 140, Strand, with the words, "Students' Column," written in the left hand top corner of the envelope.]

### APPOINTMENTS.

C. F. Beadles, appointed Assistant Medical Officer to Colney Hatch Asylum. R. Bevan, L.R.C.P., M.R.C.S., appointed Medical Officer of Health for Ashford, Kent. J. H. Clayton, M.B.Lond., L.R.C.P., M.R.C.S., appointed Casualty Surgeon to the Queen's Hospital, Birmingham. E. H. Colbeck, B.A., M.B., B.C.Cantab., M.R.C.P.Lond., appointed Assistant Physician to the City of London Hospital for Diseases of the Chest, Victoria Park, E. J. C. H. Dickinson, M.B.Camb., L.S.A., appointed Medical Officer of the Workhouse of the Stepney Union. G. F. Duffey, M.D.Dub., F.R.C.P.I., appointed Inspector of Examinations for the General Medical Council. T. Geraty, M.R.C.S.Eng., L.R.C.P.I., appointed Honorary Consulting Surgeon to the Nottingham General Dispensary. F. A. Humphry, F.R.C.S., appointed Honorary Consulting Surgeon to the Sussex County Hospital. W. J. McKay, M.B., M.Ch., B.Sc.Sydney, appointed Resident Medical Officer to the Soho Hospital for Women. C. H. D. Morland, M.B., B.A.Durh., L.R.C.P.Lond., M.R.C.S., appointed House Surgeon to the Cardiff Infirmary. E. Morris, M.R.C.S., appointed Honorary Surgeon to the Hereford General Infirmary. J. Phillips, M.A., M.D.Cantab., M.R.C.P., appointed Examiner in Midwifery to the Glasgow University. E. S. Reynolds, M.D.Lond., M.R.C.P., M.R.C.S., appointed Medical Officer of the Crumpsall Workhouse, Township of Manchester. W. Richards, appointed Honorary Dental Surgeon to Fowey Cottage Hospital. F. T. Roberts, M.D., F.R.C.P.Lond., appointed Consulting Physician to the Hospital for Consumption, Brompton. W. J. Turrell, M.A., M.B., B.Ch.Oxon., appointed Medical Officer to the Cutler Boulter Provident Dispensary, Oxford.

### VACANCIES.

### Resident Medical Officers.

Bedford General Infirmary, Surgeon, doubly qualified—Salary £100, &c.
Camberwell House Asylum, Camberwell, S.E., Junior Medical Officer; unmarried—Salary £100, &c.
Chorley General Dispensary, House Surgeon and Apothecary, doubly qualified—Salary £120, &c.
City of Liverpool Infectious Diseases Hospitals, Medical Officers for the City Hospital North, Netherfield Road, and City Hospital South, Grafton Street, doubly qualified, and not more than 30 years of age—Salary £100 per annum, increasing annually to £120, &c.
City of London Hospital for Diseases of the Chest, Victoria Park, E., House Physician—Board, &c.
County Asylum, Whittingham, Preston, Assistant Medical Officer—Salary £150, &c.
Denbighshire Infirmary, Denbigh, House Surgeon. Must be conversant with the Welsh language—Salary £85, &c.
East Suffolk and Ipswich Hospital, Thoroughfare, Ipswich, House Surgeon, unmarried, doubly qualified—Salary £100, &c.
Exeter City Asylum, Digby, near Exeter, Assistant Medical Officer, unmarried—Salary £100, &c., rising £10 annually to £150.
Hoxton House Asylum, N., Assistant Medical Officer—Salary £120, &c., rising £10 a year to £160.
Leeds Public Dispensary, Medical Officer—Salary £85.
Lincoln County Hospital, House Surgeon, doubly qualified, unmarried, and under 40 years of age—Salary £100, &c.
Manchester Southern and Maternity Hospital, House Surgeon.
North-Eastern Hospital for Children, Hackney Road, N.E., Junior House Surgeon for nine months, doubly qualified—Salary at the rate of £60, &c.
Royal Berks Hospital, Reading, Assistant House Surgeon—Salary £40, &c. Appointment for six months.
Sheffield Union, Assistant Medical Officer for the Workhouse at Fir Vale, Pitsmoor—Salary £100, &c.
St. Mark's Hospital for Fistula, &c., City Road, E.C., House Surgeon—Salary £50, &c. Appointment for twelve months.
West London Hospital, Hammersmith Road, W., House Physician—Board, &c. Appointment for six months.
West London Hospital, Hammersmith Road, W., House Surgeon—Board, &c. Appointment for six months.

### Non-Resident Medical Officers.

Dental Hospital of London, Leicester Square, two Assistant Dental Surgeons; must be Licentiates in Dental Surgery.
Dental Hospital of London and London School of Dental Surgery, Leicester Square, Demonstrator—Honorarium, £50.
London Lock Hospital and Asylum, Harrow Road, W., and 91, Dean Street, Soho, W.—Registrar.
London Temperance Hospital, Hampstead Road, N.W., Physician, doubly qualified.
London Temperance Hospital, Hampstead Road, N.W., Assistant Physician, doubly qualified.

### The Hospital Advertiser.

### MIDDLESEX HOSPITAL MEDICAL SCHOOL.

### THE STUDENT OF MEDICINE—(*continued*).

### NOTES FROM THE SCHOOLS.

### Edinburgh University.

At the annual general meeting of the Edinburgh University Cycling Club the following office-bearers for 1892 were elected: President, J. H. A. Laing, M.B.; vice-president, A. Cameron; captain, J. M. Taylor; sub-captains, F. J. Hare, B.Sc., and C. H. Adamson; hon. secretary, C. A. Edmondson.

In the last examination for the Indian Medical Service the following Edinburgh graduates head the list: 1. P. B. Haig, late resident surgeon Edinburgh Royal Infirmary, resident at the Sick Children's Hospital, and ex-President of the Royal Medical Society. 2. C. Robson Scott, late house physician City of London Hospital. 3. H. G. Melville, Demonstrator of Anatomy in the University, late resident physician R.E.I., late President of the Union and of Students' Representative Council; also colour-sergeant in the University Company of the Queen's Edinburgh Rifle Brigade. 4. R. H. Maddon, late resident at the Royal Maternity Hospital, Edinburgh. Mr. J. L. Macrae is also on the list.

The Athletic Club held their boxing championships on February 20. Results: Feather weights, Wilson; light weights, undecided; middle weights, Sibbald; heavy weights, "Hardress Cregan."

Professor Ferrier delivered an address on "Cerebral Localisation in Relation to Therapeutics," to graduates and students of the University, on February 26. In the evening Dr. Ferrier was the guest of the Royal Medical Society at their annual dinner.

### PASS LISTS, Etc.

### Society of Apothecaries of London.

The following candidates passed in:

SURGERY.—R. Freer, St. Mary's Hospital; A. W. Fyffe, Middlesex Hospital and Belfast; W. C. Hinde, Middlesex Hospital; J. H. Hobling, St. Mary's Hospital; H. F. Ransome, Manchester (Owens College); F. K. Rider, Leeds (Yorkshire College); J. W. Smith, London Hospital and Manchester; P. M. Toms, Middlesex Hospital.

MEDICINE, FORENSIC MEDICINE, AND MIDWIFERY.—G. H. Baird, St. Mary's Hospital and Dublin; W. T. B. Donnelly, Guy's Hospital; W. Overend, St. Bartholomew's Hospital; D. L. Thomas, London Hospital.

MEDICINE AND MIDWIFERY.—G. M. Arkle, Liverpool Royal Infirmary; V. J. R. Robin, King's College; G. P. Smith, Guy's Hospital; H. S. Ware, St. Thomas's Hospital.

FORENSIC MEDICINE.—P. J. Naden, Birmingham (Queen's College).

MIDWIFERY.—G. R. S. Breeze, Royal Free Hospital; J. R. Daly, King's College.

To Messrs. Arkle, Baird, Daly, Hinde, Overend, and Miss Breeze was granted the diploma of the Society entitling them to practice medicine, surgery, and midwifery.

### Army Medical Staff.

The following is the list of successful candidates for commissions in the Medical Staff of Her Majesty's Army at the recent examination in London:—

| | Marks. | | Marks. |
|---|---|---|---|
| Hinge, H.A. ... ... | 3,160 | Jones, T. P. ... ... | 2,830 |
| McDermott, T. ... ... | 3.160 | Marder, N. ... ... | 2,810 |
| Bray, H. A. ... ... | 3,140 | Thurston, H. S.... ... | 2,810 |
| Hodgens, C. O'C. ... | 3,095 | Lewis, R. C. ... ... | 2,795 |
| Slayter, E. W. ... ... | 3,070 | More, L. P. ... ... | 2,790 |
| Erskine, W. D. ... ... | 3,020 | Faichnie, F. ... ... | 2,760 |
| Tyrrell, A. F. ... ... | 2,945 | Walker, G. S. ... ... | 2,730 |
| McNaught, J. G. ... | 2,940 | O'Reilly, W. H. ... ... | 2.710 |
| Smyth, W. J. ... ... | 2,940 | Mansfield, G. S. ... ... | 2,705 |
| Chambers, J. G.... ... | 2,910 | Ryall, W. P. ... ... | 2,695 |
| Austin, R. F. E. ... ... | 2.900 | Thompson. A. G. ... | 2,670 |
| Moore, G. A. ... ... | 2,900 | Read, H. W. K. ... ... | 2,665 |
| Condon, E. H. ... ... | 2,870 | | |

### Indian Medical Service.

The following is a list of successful candidates for the Indian Medical Service at the competitive examination held on February 8th and the following days:—

| | Marks. | | Marks. |
|---|---|---|---|
| Haig, P. B. ... ... | 3,425 | Swinton, F. E. ... ... | 3,230 |
| Robson-Scott, G.C. ... | 3,395 | Green, D. R. ... ... | 3,195 |
| Melville, H. G. ... ... | 3,370 | Smith, G. M'I. C. ... | 3,155 |
| Maddox, R. H. ... ... | 3,310 | Burnett, S. H. ... ... | 3,145 |
| Earle, H. M. ... ... | 3,290 | Jackson, T. ... ... | 3,145 |
| Fullerton, T. W. A. ... | 3,260 | Hulbert, J. G. ... ... | 3,140 |
| Hugo, E. V. ... ... | 3,250 | Macrae, J. L. ... ... | 3,065 |
| Hubbard, A. O. ... ... | 3,245 | Gabbett, P. C. ... ... | 3,060 |
| Smith, H. A. ... ... | 3,235 | | |

Forty-seven candidates competed for seventeen appointments, and all were reported qualified.

### ATHLETICS.

### Football.—RUGBY.

INTER-HOSPITAL CHALLENGE CUP (SEMI-FINAL).

ST. BARTHOLOMEW'S *v.* ST. GEORGE'S.—Won by St. Bartholomew's, who had the best of the game throughout, by two goals and two tries (14 points) to nil.

ASSOCIATION (SEMI-FINAL).

ST. MARY'S *v.* ST. THOMAS'S.—St. Mary's won by two goals to one.

